# A Sheep Behavior Recognition Approach Based on Improved FESS-YOLOv8n Neural Network

**DOI:** 10.3390/ani15060893

**Published:** 2025-03-20

**Authors:** Xiuru Guo, Chunyue Ma, Chen Wang, Xiaochen Cui, Guangdi Xu, Ruimin Wang, Yuqi Liu, Bo Sun, Zhijun Wang, Xuchao Guo

**Affiliations:** 1 College of Information Science and Engineering, Shandong Agricultural University, Taian 271018, China; gxr1662@163.com (X.G.); mmccdyx@163.com (C.M.); vanchn14@163.com (C.W.); cxc20020429@163.com (X.C.); xud23112@163.com (G.X.); wangruimin0202@163.com (R.W.); liuyuqi2600@163.com (Y.L.); sunbo87@126.com (B.S.); 2 Apple Technology Innovation Center of Shandong Province, Taian 271018, China

**Keywords:** sheep, behavior recognition, object detection, YOLOv8, lightweight

## Abstract

Sheep are an important breed of livestock in the northern regions of China, providing humans with nutritious meat and by-products. Therefore, it is essential to ensure the health status of sheep. Research has shown that the individual and group behaviors of sheep can reflect their overall health status. However, as the scale of farming expands, traditional behavior detection methods based on manual observation and those that employ contact-based devices face challenges, including poor real-time performance and unstable accuracy, making them difficult to meet the current demands. To address these issues, this paper proposes a sheep behavior detection model, Fess-YOLOv8n, based on an enhanced YOLOv8n neural network. On the one hand, this approach achieves a lightweight model by introducing the FasterNet structure and the selective channel down-sampling module (SCDown). On the other hand, it utilizes the efficient multi-scale attention mechanism (EMA)as well as the spatial and channel synergistic attention module (SCSA) to improve recognition performance. The results on a self-built dataset show that Fess-YOLOv8n reduced the model size by 2.56 MB and increased the detection accuracy by 4.7%. It provides technical support for large-scale sheep behavior detection and lays a foundation for sheep health monitoring.

## Simple Summary

This study proposes an efficient sheep behavior recognition method, FESS-YOLOv8n, which aims to accurately identify the activity, eating, and lying behaviors of sheep by integrating deep learning and computer vision technologies while also monitoring their health and enabling timely preventive measures. Experimental results demonstrate that the proposed method significantly enhances the accuracy of sheep behavior recognition while achieving a lightweight model. This method provides precise behavioral recognition and physiological health assessment tools for the livestock industry, thereby facilitating the development of large-scale farming and the modernization of sheep farming practices.

## 1. Introduction

Native to central and southwestern Asia and Europe, *Ovis aries*, commonly known as sheep, is now widely raised in northern China [1]. It is a high-quality breed characterized by strong adaptability, tender meat, and fine wool, offering multiple economic benefits including meat, wool, and leather. In recent years, with the rapid development of sheep farming within the livestock industry, the stocking density has rapidly increased [2,3]. High stocking density leads to limited space for activity, which weakens their immunity and affects the health status of sheep. Studies have shown that the behaviors exhibited by sheep during their activity are an important indicator of their adaptability and health condition [4,5,6]. Typically, sheep exhibit normal, common behaviors such as lying, walking, eating, and drinking. When their living environment or conditions change, abnormal behaviors such as excessive lying, reduced activity, loss of appetite, lameness, and frequent vocalizations may occur. Therefore, accurately identifying the behavioral activities of sheep is a significant method for monitoring their health status and an essential aspect of health management and control [7,8,9].

Traditional livestock monitoring which mainly relies on the installation of surveillance cameras to manually observe animal behaviors, involves issues including high workload, subjectivity, and poor real-time performance. To overcome these limitations, researchers have achieved the identification and monitoring of common livestock behaviors by using wearable devices, including triaxial accelerometers and triaxial gyroscopes [10,11,12,13,14,15]. Yin et al. [16] installed wireless sensor nodes on the necks of dairy cows and used the K-means clustering algorithm to accurately monitor parameters like respiration rate, activity acceleration, and others to assess the cows’ health status. Alvarenga et al. [17] proposed a method for recognizing sheep’s eating behavior based on a triaxial accelerometer. This method effectively distinguishes between biting and chewing behaviors by installing the accelerometer on the sheep’s jaw and using a decision tree algorithm. Zhang et al. [18] designed a wireless data acquisition system based on a triaxial accelerometer and applied deep learning models to achieve high-precision recognition of the eating, chewing, and rumination behaviors of grazing sheep. Nasirahmadi et al. [19] used object detectors to recognize pigs’ standing, lateral, and prone postures. Lee et al. [20] utilized a Kinect sensor to collect depth information and applied support vector machines to detect aggressive behavior in pigs. Yan et al. [21] selected the MPU6050 and Bluetooth transmission module as an integrated behavior data acquisition module to classify and recognize the standing, lateral, and tilted postures of sows. Although these methods have achieved good results in behavior recognition, the use of contact-based devices may restrict livestock movement and affect their daily life. In addition, the devices need to be placed accurately on specific parts of the livestock, which presents limitations in terms of their general applicability.

Deep learning, due to its end-to-end nature and the advantage of not requiring manual feature extraction, makes it a high-performance algorithm [22]. In the field of livestock monitoring, it offers a cost-effective, low-cost, non-contact approach for livestock behavior recognition [23,24,25,26,27]. Wang et al. [28] proposed a lightweight behavior recognition model for dairy goats, GSCW-YOLO, based on YOLOv8n. By integrating Gaussian Context Transformation and Content-Aware Reassembly of Features, the model enhances behavior feature recognition accuracy and small target detection capabilities, enabling automatic identification of abnormal behaviors in dairy goats. To achieve real-time online recognition of Liaoning cashmere goat behaviors, Chen et al. [29] developed a high-precision and efficient behavior recognition model based on the YOLOv8n lightweight object detection algorithm. The model utilizes data augmentation, the CBAM attention mechanism, and Alpha-CIOU to improve recognition performance. Hao et al. [30] proposed the YOLOv5-EMA model, which introduces an efficient multi-scale attention module that significantly improves the detection accuracy of cattle bodies and key parts, especially in the presence of small targets and occlusion. Yu et al. [31] introduced the Res-Dense YOLO model for recognition of daily behavior in dairy cows, based on the YOLOv5 framework. This model incorporates multi-scale detection heads, the CoordAtt attention mechanism, and SioU loss function to enhance the recognition accuracy of behaviors such as drinking, feeding, lying, and standing in dairy cows. Wang et al. [32] optimized the sheep behavior recognition model based on YOLOv8s, making improvements in small object detection, model lightweighting, and other aspects, achieving accurate recognition of behaviors such as standing, walking, eating, drinking, and lying. Song et al. [33] proposed the ECA-YOLOv5s behavior recognition model, based on the YOLOv5s network and a channel-wise attention module, which enhances the recognition accuracy and stability of behavior in beef cattle under complex occlusion and varying lighting conditions. Yang et al. [34] adopted Dense Block and SPPCSPC modules on the YOLOv6 framework to improve the recognition accuracy of abnormal pecking and pecked behaviors in chickens, facilitating the intelligent detection of abnormal behaviors in laying hens. Duan et al. [35] employed a lightweight network structure and attention modules to develop a behavior recognition method for beef cattle based on SNSS-YOLOv7. This method reduces the computational load while accurately identifying common cattle behaviors. Gao et al. [36] proposed a multi-scale behavior recognition method for dairy cows based on an improved YOLOv5s network, which enhances the recognition accuracy of daily behaviors, including standing, drinking, walking, and lying. Li et al. [37] introduced a mounting behavior recognition algorithm for pigs based on Mask R-CNN, which automatically detects mounting behavior in pigs. Ding et al. [38] achieved precise detection of suckling piglets by quantifying and optimizing the YOLOv5 network and efficiently deployed the model on the Jetson Nano platform.

Comprehensive analysis shows that, compared to contact-based devices for livestock behavior recognition, non-contact recognition methods have advantages such as being non-destructive, stress-free, cost-effective, and less affected by environmental factors. However, such research is still in its early stages, and current technologies are primarily focused on simple backgrounds. The effectiveness and accuracy of these methods need to be further improved in high-density and complex backgrounds. Therefore, this paper focuses on three common sheep behaviors: activity, eating, and lying, while also proposing an improved YOLOv8n-based model for sheep behavior recognition, called Fess-YOLOv8n. Firstly, the C2f module in the YOLOv8n backbone network replaces C2f-Faster to mitigate the computational load and reduce the model’s parameter size. Secondly, to address the issue of weak feature extraction due to occlusion of the sheep or external environmental factors, an efficient multi-scale attention module (EMA) is introduced. In addition, a spatial-channel synergistic attention mechanism (SCSA) is implemented to allocate appropriate weights to the model’s spatial and channel features, thereby enhancing its ability to fuse and detect targets across different scales. Finally, selective channel down-sampling (SCDown) is incorporated into the model, utilizing point convolutions and depth convolutions to adjust channel dimensions and spatial resolution, respectively, making the model lightweight while enhancing detection accuracy. The main contributions of this paper are as follows:

(1) Constructing a sheep behavior dataset and proposing the Fess-YOLOv8n model for sheep behavior recognition, which strikes a balance between lightweight design and high-precision recognition. Specifically, the C2f-Faster and SCDown modules contribute to the lightweight design by reducing computational complexity and parameter size, while the integration of EMA and SCSA improves recognition accuracy by enhancing feature extraction.

(2) Investigating the effects of different IoU thresholds, optimizers, and learning rates on Fess-YOLOv8n model training performance and behavior recognition effectiveness.

(3) Benchmarking the proposed model’s performance against other classical deep learning models on sheep behavior recognition tasks.

## 2. Materials and Methods

### 2.1. Dataset Construction

**Figure 1:**
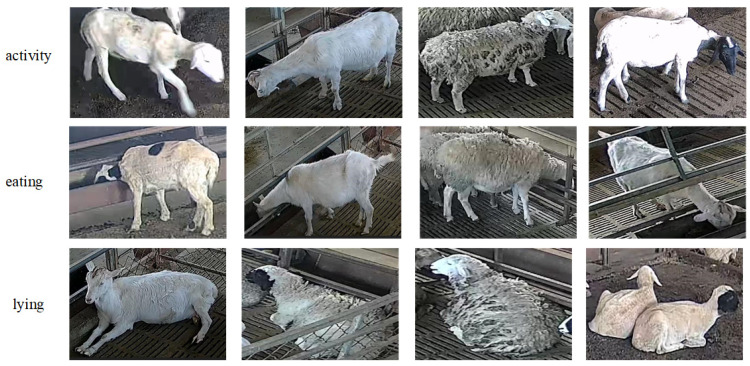
Examples of different sheep behaviors.

**Figure 2:**
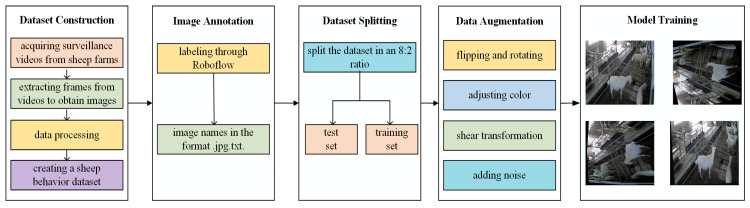
Process of constructing the sheep behavior dataset.

The data used in this study were collected from a sheep farm in Shankou Town, Taian City, Shandong Province, between April and May 2024. The collection device was a jovision technology camera (Jinan, Shandong, China, resolution: 1920 × 1080 pixels, video frame rate: 20 fps). Compared to nighttime, sheep exhibit higher activity levels under better lighting conditions during the day. Therefore, the video collection period was set from 09:00 to 17:00 to ensure both image quality and the diversity of sheep behaviors. A total of 1200 daily activity videos of sheep were collected. To enhance the behavioral diversity between images and reduce data redundancy, one frame was extracted every 50 frames from each video, resulting in a total of 1915 sheep behavior images. The dataset mainly includes *Ovis aries*, a common species of sheep, but the sample does not limit gender or age, including both male and female sheep from lambs to adult sheep. As shown in Figure 1, this study focused on three common sheep behaviors, including activity, eating, and lying. The sheep behavior categories and their corresponding labels are shown in Table 1. The process of constructing the sheep behavior dataset is illustrated in Figure 2. All images were labeled using Roboflow 1.0 and the dataset was randomly divided into a training set and a test set with a ratio of 8:2.

### 2.2. Data Augmentation

**Figure 3:**
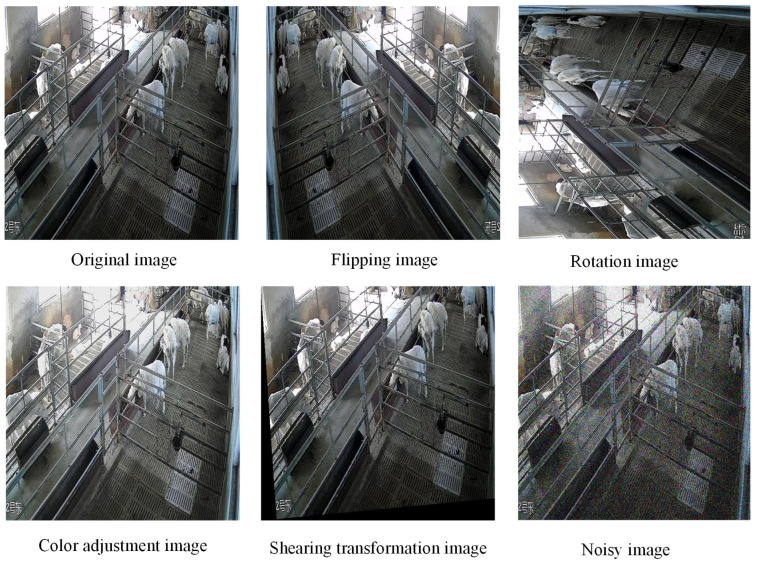
Sheep behavior images with data augmentation.

To increase image diversity and enhance the model’s generalization ability, image augmentation techniques were applied. The specific techniques included flipping, rotation, color adjustment, shearing transformation, and the addition of noise, simulating various shooting angles, lighting conditions, and environmental changes. As a result, the dataset size was expanded from 1915 images to 4979 images. The augmented sheep behavior images are shown in Figure 3. After data augmentation, the label counts for the behavior categories activity, eating, and lying were 8082, 14,144, and 5321, respectively.

### 2.3. Improved YOLOv8n-Based Detection Model

YOLOv8 builds on the efficiency and real-time performance of the YOLO series, with several structural optimizations that enhance detection accuracy and processing speed [39]. YOLOv8 is mainly divided into three components: Backbone, Neck, and Head, which are responsible for feature extraction, feature fusion, and final output generation, respectively. For further details on its basic structure, refer to the literature by Terven et al. [39]. The algorithm offers multiple versions (n, s, m, l, x), among which the YOLOv8n model, with lower complexity and higher computational efficiency, is better suited for lightweight and high-speed applications. Therefore, this study uses YOLOv8n as the base model and further improves upon it.

**Figure 4:**
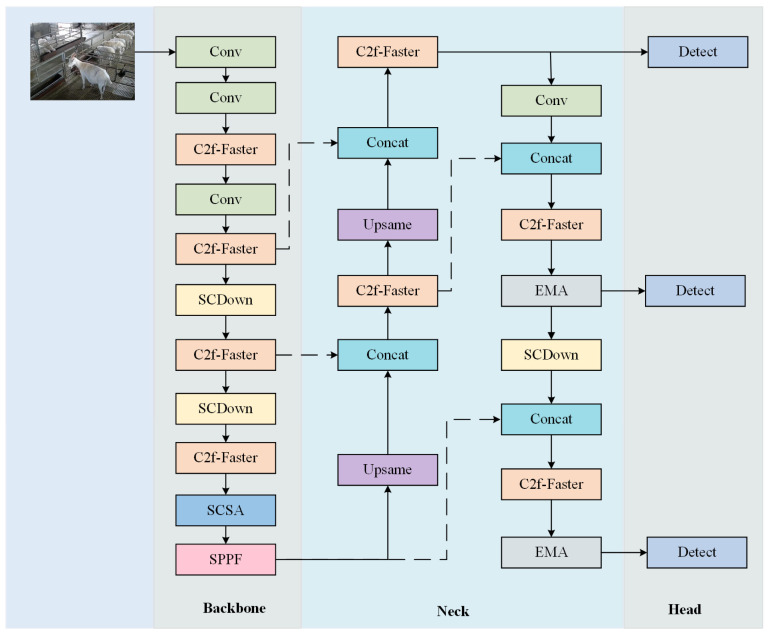
Architecture of the Fess-YOLOv8n model.

In the sheep behavior detection task, due to the complex environment (e.g., intricate farming conditions, diverse sheep behaviors, severe occlusion, and strong background interference), existing models struggle with recognizing sheep behaviors and dealing with occlusion and complex backgrounds. To tackle these challenges, we propose an improved model based on YOLOv8n, called Fess-YOLOv8n. The architecture of the Fess-YOLOv8n network is illustrated in Figure 4. Firstly, FasterNet is used to replace the original network structure and reduce the model’s parameter count and computational costs. Next, the EMA attention mechanism is introduced to strengthen key feature information and improve the model’s feature representation capability. Subsequently, the SCSA attention mechanism is added to exploit the synergistic effects between channel and spatial attention, thereby enhancing detection accuracy. Finally, SCDown is employed to reduce the model’s computational load and complexity while ensuring detection accuracy.

#### 2.3.1. Lightweighting Model Networks

- C2f-Faster Module

**Figure 5:**
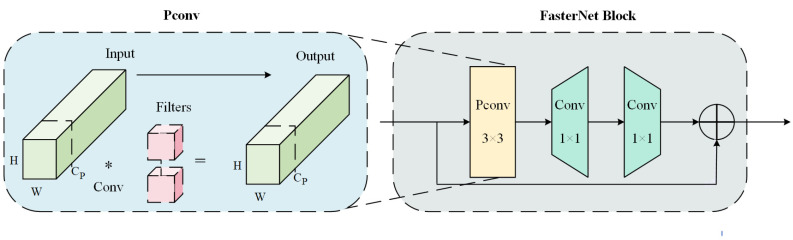
Structure of the FasterNet block.

Compared to other modules in the YOLOv8 series, YOLOv8n has fewer parameters. However, when dealing with complex tasks, it still faces a significant computational burden and complexity, especially in the task of sheep behavior recognition, where image data extracted from video streams need to be processed. This increases the demand for the model’s detection speed. To address this, the study adopts the C2f framework and replaces the Bottleneck with the FasterNet Block, constructing the C2f-Faster module [40]. Compared to the original module, it effectively reduces the computational burden of the FasterBlock structure and shortens the detection time. Specifically, the FasterNet block consists of partial convolution (PConv) and two pointwise convolution layers. PConv performs convolutions only within selected regions to reduce redundant computations and memory accesses. The pointwise convolution layers adjust the number of channels through convolution, optimizing feature representation capability. The FasterNet structure is illustrated in Figure 5, and the floating-point operations per second (FLOPS) for regular convolution and PConv are presented in Equations (1)–(3):

(1) $$F=H\times W\times K^{2}\times C$$

(2) $$F_{P}=H\times W\times K^{2}\times C_{P}^{2}$$

(3) $$R=\frac{C_{P}}{C}$$

where K denotes the size of the convolution kernel; C, H and W represent the number of channels, height, and width of the feature map, respectively; $C_{P}$ is the number of channels for regular convolution features; and R is the reduction factor.

- SCDown Module

Traditional deep learning models face the challenges of high complexity and slow inference speed, especially when processing high-resolution images, where detailed features add to the computational burden and slow down sheep behavior recognition. To address this issue, the Fess-YOLOv8n model introduces the SCDown module [41]. This module uses pointwise convolution to adjust the number of input feature channels, avoiding the increasing computational costs that arise when traditional convolutions expand the number of channels. Subsequently, depthwise separable convolutions are employed for spatial down-sampling, splitting the traditional convolution operation into depthwise and pointwise convolutions. Each part handles a distinct computational task: depthwise convolutions independently apply kernels to each input channel, avoiding cross-channel interaction, while pointwise convolutions fuse the output channel information from the depthwise convolutions to generate new channel features. The computational complexity of SCDown is shown in Equations (4)–(7):

(4) $${Flops}_{1\times 1}=H\times W\times C_{in}\times C_{out}$$

(5) $${Flops}_{\mathrm{deptwise}}=H_{\mathrm{out}}\times W_{out}\times K\times K\times C_{out}$$

(6) $${Flops}_{\mathrm{pointwise}}=H_{\mathrm{out}}\times W_{out}\times C_{out}^{2}$$

(7) $${Flops}_{ScDdown}{=Flops}_{1\times 1}+{Flops}_{\mathrm{deptwise}}+{Flops}_{\mathrm{pointwise}}$$

where H×W represents the spatial dimensions of the input feature map, $C_{in}$ and $C_{out}$ denote the number of input and output channels, respectively; and K×K represents the size of the convolution kernel.

#### 2.3.2. Enhancing Feature Extraction and Fusion

- EMA Module

**Figure 6:**
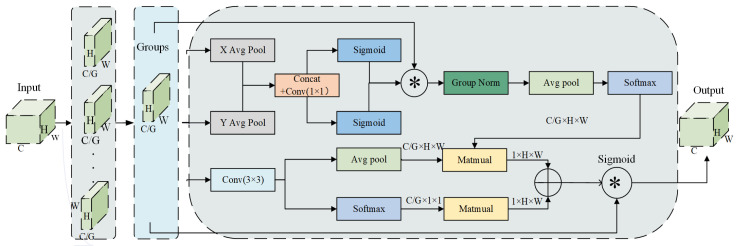
Structure diagram of EMA.

Sheep behavior is inherently complex and unpredictable. For instance, during sheep running, the rapid movement of limbs and dynamic changes in body posture led to significant variations. Enhancing the model’s ability to extract key features is a primary concern. Additionally, complex backgrounds and environmental interference can cause the model to rely on local information, leading to the loss of crucial features and impacting overall recognition performance. In response to these difficulties, Fess-YOLOv8n utilizes the EMA module [42], which enhances the model’s ability to extract key features by leveraging dual attention mechanisms. The structure of the EMA module is illustrated in Figure 6. The implementation process is as follows: Firstly, the input feature map C×H×W is grouped, where C represents the number of channels, and H and W are the height and width of the feature map, respectively. Through the operation of grouped convolutions, the channels are divided into G groups, generating an output feature map of shape C×H×W/G. Then, through 1×1 and 3×3 convolution branches, features are extracted along the channel and spatial dimensions, respectively. In the 1×1 convolution branch, each group of feature maps undergoes global average pooling (AvgPool) along the X and Y dimensions to extract global features, allowing the model to focus on recognizing key features of sheep behavior, such as those containing information about the legs or body posture during rapid movement. The pooled results are then concatenated along the spatial dimension to form a new feature map. Subsequently, 1×1 convolution operation is applied to the resulting feature map, followed by the Sigmoid activation function to generate attention weights, ensuring the weights are within the range of [0, 1]. The processed feature map is then normalized using Group Normalization (GN) [43] to ensure numerical stability. Global features are extracted through AvgPool, and Softmax is applied to generate the channel attention map. In the 3×3 convolution branch, the feature space is processed through convolution to capture more extensive spatial information. Following this, operations such as AvgPool and Softmax are performed to generate the spatial attention map, which not only enhances the focus on key features but also enables the model to effectively locate and highlight important areas related to sheep movement. Finally, the channel attention map and the spatial attention map are fused through addition to integrate feature information from different scales. The fused attention map is processed by the Sigmoid activation function and applied to the original feature map via element-wise multiplication, resulting in the optimized feature map. This dual attention mechanism ensures that the model captures both the most important channels and the most relevant spatial regions, improving its ability to recognize sheep behavior in dynamic and complex environments.

- SCSA Module

**Figure 7:**
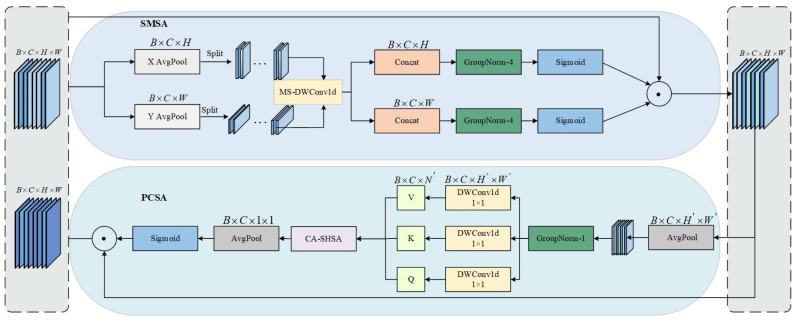
Structure diagram of SCSA.

In sheep behavior recognition, the behavioral changes are often irregular, with these changes primarily concentrated on local features, making it difficult to effectively integrate global spatial information. As a result, this limits the model’s ability to recognize the overall structure and key relationships of sheep behavior. To tackle the issues mentioned above, Fess-YOLOv8n incorporates the SCSA module into the Backbone, which decomposes the attention mechanism across both spatial and channel dimensions. This allows the model to fully utilize the inherent multi-semantic spatial information, extracting key features from both the spatial and channel dimensions while reducing irrelevant features, thus enhancing model accuracy. SCSA consists of two components: Shareable Multi-Semantic Spatial Attention (SMSA) and Progressive Channel-wise Self-Attention (PCSA) [44]. SMSA effectively captures the spatial dependencies of sheep behavior under different conditions, especially when behavioral changes are significant, by identifying the spatial structural changes in full-body behavior. By integrating multi-level semantic information, SMSA enables accurate recognition of full-body behavior even under complex behavioral variations and occlusions. PCSA optimizes channel features through an input-aware self-attention mechanism, addressing feature discrepancies between channels caused by behavioral changes, thereby enhancing the model’s adaptability to these variations and improving its robustness to irregular behaviors. The structure of SCSA is illustrated in Figure 7.

The tensor B×C×H×W is decomposed into two unidirectional 1D sequences, B×C×W and B×C×H, and global average pooling is applied along each dimension. Then, the feature set is divided into independent sub-features of size k, denoted as $X_{H}^{i}$ and $X_{W}^{i}$, which undergo depthwise 1D convolution and lightweight shared convolution operations to explore the diversified spatial structures between features and enrich semantic information. The formulas for the decomposition of the feature map and the extraction of multi-semantic spatial information are as follows:

(8) $$X_{H}^{i}=X_{H}\left[:,\left(i-1\right)\times \frac{C}{K}:i\times \frac{C}{K},:\right]$$

(9) $$X_{W}^{i}=X_{W}\left[:,\left(i-1\right)\times \frac{C}{K}:i\times \frac{C}{K},:\right]$$

(10) $$X_{H}^{∼i}=DWConv{1d}_{k_{i}}^{\frac{C}{K}\to \frac{C}{K}}\left(X_{H}^{i}\right)$$

(11) $$X_{W}^{∼i}=DWConv{1d}_{k_{i}}^{\frac{C}{K}\to \frac{C}{K}}\left(X_{w}^{i}\right)$$

where $X^{i}$ denotes the i-th sub-feature, $\left(i\in \left[1,K\right]\right)$ represents the spatial structure information of the i-th sub-feature obtained after the lightweight convolution operation, and $k_{i}$ indicates the convolution kernel applied to the i-th sub-feature.

Subsequently, the semantic sub-features are concatenated and normalized, and the Sigmoid activation function is applied to enhance or suppress the activity of specific spatial regions, completing the construction of SMSA. Next, the PSCA module is constructed to alleviate the semantic discrepancies caused by multi-scale convolutions. To accurately compute the similarity between different channels, single-head self-attention (SHSA) is combined with SMSA. Additionally, a progressive compression method based on average pooling is used to reduce the computational costs of SHSA while preserving the semantic information extracted by SMSA. The construction formulas of SCSA are as follows:

(12) $${Attn}_{H}=\sigma (GN_{H}^{K}(Concat({X^{∼}}_{H}^{1},{X^{∼}}_{H}^{2},\ldots ,{X^{∼}}_{H}^{K})))$$

(13) $${Attn}_{W}=\sigma (GN_{W}^{K}(Concat({X^{∼}}_{W}^{1},{X^{∼}}_{W}^{2},\ldots ,{X^{∼}}_{W}^{K})))$$

(14) $$SMSA(X)=X_{S}={Attn}_{H}\times {Attn}_{w}\times X$$

(15) $$X_{P}={Pool}_{\left(7,7\right)}^{\left(H,W\right)\to \left(H^{\prime },W^{\prime }\right)}\left(X_{s}\right)$$

(16) $$F_{proj}=DWConv1d_{\left(1,1\right)}^{C\to C}$$

(17) $$Q=F_{proj}^{Q}\left(X_{P}\right),K=F_{proj}^{K}\left(X_{P}\right),V=F_{proj}^{V}\left(X_{P}\right),$$

(18) $$X_{attn}=Attn\left(Q,K,V\right)=Soft\max\left(\frac{QK^{T}}{\sqrt{C}}\right)V$$

(19) $$PCSA\left(X_{s}\right)=X_{c}=X_{s}\times \sigma \left(Pool_{\left(H^{\prime },W^{\prime }\right)}^{\left(H^{\prime },W^{\prime }\right)\to \left(1,1\right)}\left(X_{attn}\right)\right)$$

(20) $$SCSA\left(X\right)=PCSA\left(SMSA\left(X\right)\right)$$

where k×k denotes the kernel size, $\left(H,W\right)$ and $\left(H^{\prime },W^{\prime }\right)$ represent the resolution dimensions, and $F_{proj}\left(•\right)$ denotes the linear projection that generates the query, key, and value.

## 3. Results

### 3.1. Experimental Environment

The experiments were conducted using the PyTorch 2.2.0 deep learning framework. The experimental setup consisted of a 64-bit Windows 11 system with an AMD Ryzen 9 7945 HX CPU (Advanced Micro Devices, Inc., Santa Clara, CA, USA), 32 GB of RAM, and an NVIDIA GeForce RTX 4060 GPU (NVIDIA Corporation, Santa Clara, CA, USA) with 8 GB of VRAM (SK Hynix Inc., Icheon, Republic of Korea). The CUDA version was 12.1. During the training process, the number of epochs was set to 200, batch size was set to 16, and the default IoU threshold was set to 0.7. The Adam optimizer was used for network optimization, with an initial learning rate of 0.01, a momentum parameter of 0.937, and a weight decay coefficient of 0.0005.

### 3.2. Evaluation Index

This study uses several evaluation metrics, including mean average precision (mAP), average precision (AP), confusion matrix, parameter count, gigaFLOPS (GFLOPS), and weight file size, to assess the model’s performance. Specifically, AP reflects the relationship between precision (P) and recall (R), and integrates the area under the precision–recall (PR) curve to evaluate the model’s performance for each category. P measures the accuracy of positive class predictions, which is the proportion of true positive samples among all samples predicted as positive. R indicates the proportion of actual positive samples that are correctly predicted as positive by the model. mAP is calculated by averaging the AP for each class, providing an overall assessment of the model’s performance. The confusion matrix is used to represent the model’s predictions across different classes, including both correct and incorrect classifications, thus reflecting the model’s classification accuracy. The loss rate in the confusion matrix represents the proportion of misclassified samples out of all predictions, while the false positive rate represents the proportion of negative samples incorrectly predicted as positive. Additionally, parameter count, GFLOPS, and model size are used as supplementary metrics to measure the model’s computational complexity and resource requirements. The evaluation expressions for these metrics are as follows:

(21) $$P=\frac{TP}{TP+FP}$$

(22) $$R=\frac{TP}{TP+FN}$$

(23) $$AP=\int_{0}^{1}P\cdot RdR$$

(24) $$mAP=\frac{1}{N}\sum_{i=1}^{N}AP_{i}$$

where True Positive (TP) refers to the number of correctly predicted positive samples, False Positive (FP) denotes the number of incorrectly predicted positive samples, and False Negative (FN) indicates the number of incorrectly predicted negative samples. N represents the number of categories in the dataset, which is 3 in this section. The classification of positive and negative samples is determined by the Intersection over Union (IoU) between the predicted region and the actual target region in the object detection task. If the IoU between the predicted box and the ground truth box is greater than a predefined threshold, the sample is considered positive; otherwise, it is regarded as negative.

### 3.3. Ablation Experiments

**Figure 8:**
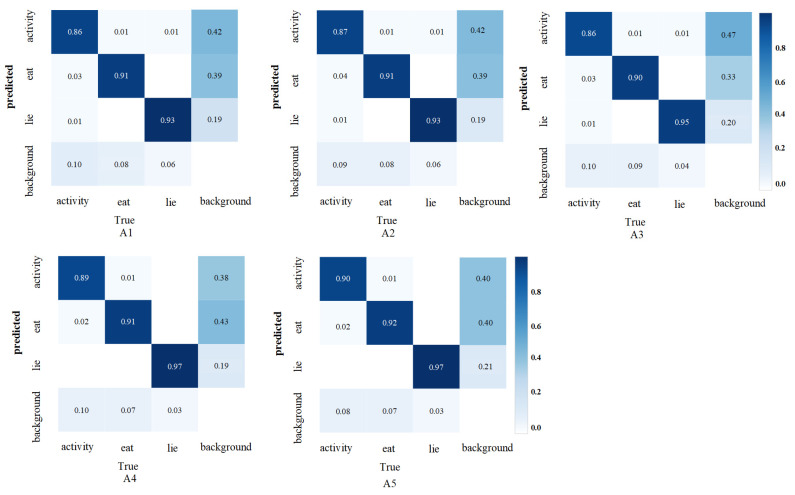
Comparison of confusion matrices across different ablation experiments.

**Figure 9:**
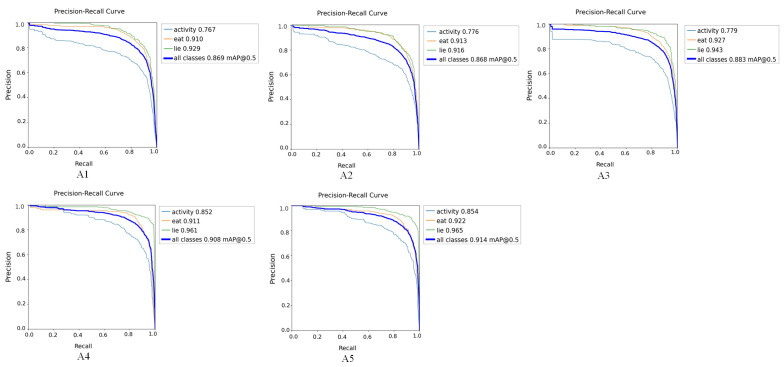
Comparison of curves across different ablation experiments.

**Figure 10:**
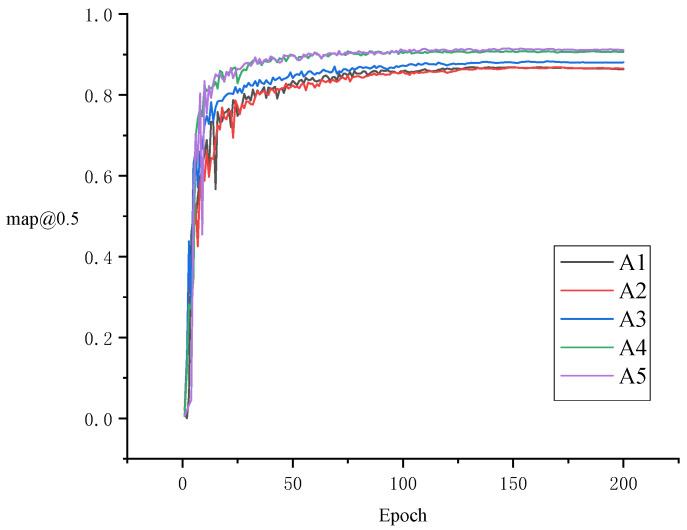
Comparison of mAP@0.5 curves across different ablation experiments.

To assess the impact of various modules on the performance of the sheep behavior recognition model, five ablation experiments were conducted using the sheep behavior dataset. Table 2 provides a comparison of the baseline model’s performance under different experimental conditions. Figure 8 compares the confusion model across various modified ablation experiments. Meanwhile, Figure 9 and Figure 10 display the curve comparison of the baseline model under different ablation experiments and the curve comparison mAP@0.5 under different ablation experiments, respectively.

Table 2 shows that the Fess-YOLOv8n model significantly reduces the weight file size from 7.69 MB to 5.13 MB relative to the YOLOv8n model. Although the floating-point operations increased from 8.2 to 16.6, the parameter count increased from 2.56 M to 3.69 M, and the model’s accuracy significantly improved. The mAP@0.5 increased by 5.2%, from 86.9% to 91.4%. The detection accuracies of the activity, eating, and lying behaviors were 85.4%, 92.2%, and 96.5%, respectively, representing improvements of 8.7%, 1.2%, and 3.6% compared to the baseline model’s accuracies of 76.7%, 91.0%, and 92.9%. Among these, the most notable improvement was observed in the detection of sheep activity, which addressed the accuracy issues of traditional detection methods for activity-related behaviors.

With the optimization of the C2F-Faster module, the model’s weight file size decreased from 7.69 MB to 4.60 MB, the parameter count decreased from 2.56 M to 2.31 M, and the floating-point operations decreased from 8.2 to 6.4, resulting in a more lightweight model. The introduction of the EMA module improved feature extraction, resulting in an increase in mAP@0.5 from 86.8% to 88.3%. Notably, the recognition accuracy for activity and lying behaviors increased to 92.7% and 94.3%, respectively. After integrating the SCSA structure into Fess-YOLOv8n, the mAP@0.5 rose to 90.8%, and the recognition accuracy for activity behavior improved by 7.3%, reaching 85.2%. The introduction of the SCDown structure further reduced the model size and improved recognition accuracy for sheep behavior detection. As a result, the mAP@0.5 increased from 90.8% to 91.4%, the weight file size decreased from 5.97 MB to 5.13 MB, the parameter size dropped from 4.01 M to 3.69 M, and the floating-point operations were reduced from 16.9 to 16.6.

**Figure 11:**
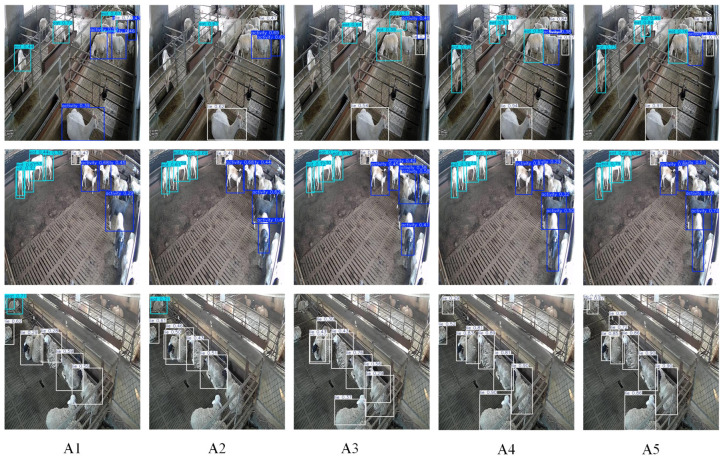
Comparison of recognition results across different ablation experiments.

Based on the comparison results presented in Figure 11, the introduction of C2F-Faster still led to misidentifications and missed detections, indicating that the method exhibits limited recognition accuracy in complex scenarios. The incorporation of the EMA module effectively reduced misidentifications, particularly when handling occluded sheep behaviors, although the confidence remained relatively low. Further integration of the SCSA and SCDown modules led to a significant improvement in overall performance.

### 3.4. Comparison of Performance at Different IoU Thresholds

**Figure 12:**
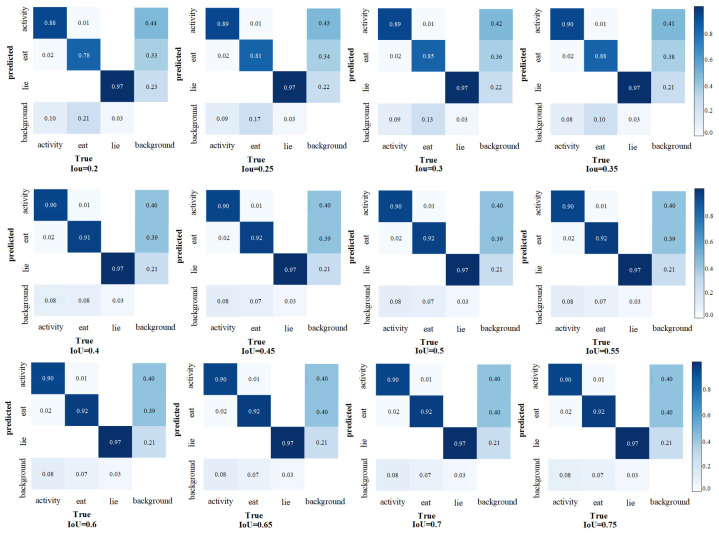
Comparison of confusion matrices at different IoU thresholds.

In this study, various Intersection over Union (IoU) thresholds were set to assess their impact on the performance of the Fess-YOLOv8n model for sheep behavior recognition. The comparative results of model performance at different IoU thresholds are shown in Table 3. Additionally, the confusion matrices for different IoU thresholds are presented in Figure 12.

From Table 3 and Figure 12, it can be observed that as the IoU threshold increases from 0.2 to 0.45, the model’s detection performance progressively improves, especially in the detection of eating behavior, where the false negative rates decreased from 21% to 7%. Moreover, the mAP@0.5 increased from 87.6% to 91.4%. For activity and lying behaviors, the variations in false positive and false negative rates remained relatively stable, with the precision consistently maintained as the IoU threshold was raised. When the IoU threshold was between 0.45 and 0.7, the false positive and false negative rates for the three behavior categories (activity, eating, lying) tended to stabilize, with false positive rates of 2%, 1%, and 0% and false negative rates of 8%, 7%, and 3%, respectively, while the mAP@0.5 remained at 91.4%.

### 3.5. Comparison of Performance Across Different Optimizers

**Figure 13:**
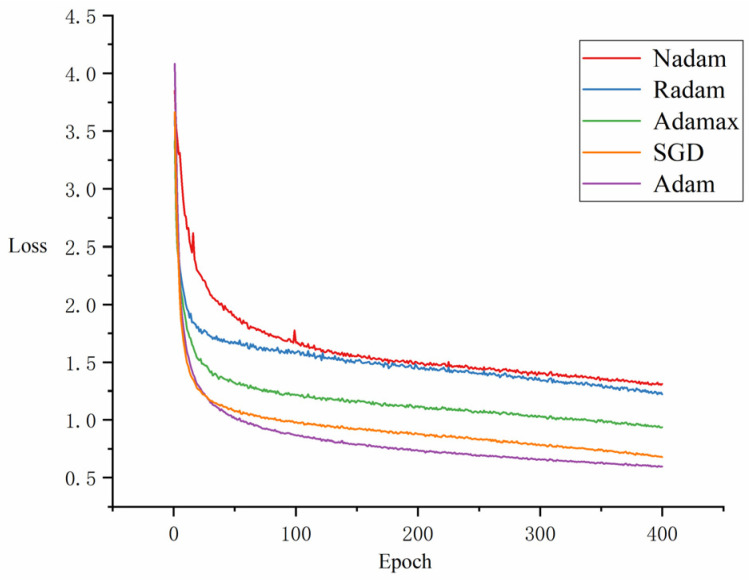
Comparison of loss curves across different optimizers.

To evaluate the influence of different optimizers on the model’s performance, experiments were conducted using a range of optimization algorithms, including Adam, Nadam, Radam, Adamax, and SGD. Table 4 presents the comparative results of model performance with each optimizer, and Figure 13 illustrates the loss curves throughout the training process for each optimizer.

From Table 4 and Figure 13, distinct performance trends can be observed for different optimizers. In terms of mAP@0.5 performance, SGD achieved the highest mAP@0.5 at 92%, closely followed by the Adam optimizer with mAP@0.5 at 91.4%, significantly outperforming Nadam, Radam, and Adamax. SGD and Adam performed very similarly, particularly in the eating and lying categories. As shown in Figure 13, the Adam optimizer exhibited a significant decrease in loss during the early stages of training. As training progressed, its loss continued to decrease steadily, ultimately reaching the lowest loss value among all optimizers. In contrast, the Nadam, Radam, and Adamax optimizers showed slower loss reduction during the initial training phase. While the SGD optimizer also demonstrated a good trend in loss reduction, it ended with a relatively higher final loss. Overall, the Adam optimizer demonstrated a good balance of fast convergence and steady performance throughout the training process.

### 3.6. Optimizing Learning Rate Performance

**Figure 14:**
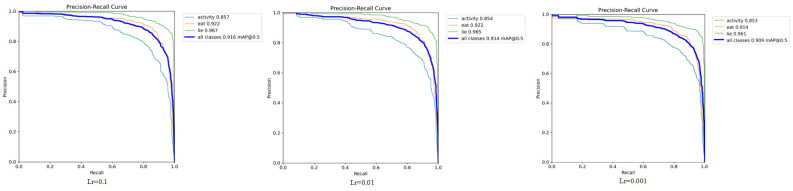
Comparison of Fess-YOLOv8n curves under different learning rates.

To optimize the model’s recognition effectiveness and select the most suitable learning rate for the Fess-YOLOv8n model tailored to this dataset, experiments were conducted to evaluate the impact of different learning rates on the model’s recognition performance. From Table 5 and Figure 14, it can be observed that the model achieved the best overall performance when the learning rate was set to 0.1, mAP@0.5 reached 91.6%, with particularly high accuracies of 85.7% for activity and 96.7% for lying. As the learning rate decreased to 0.01, overall accuracy slightly dropped, with mAP@0.5 being 91.4%. When the learning rate was 0.001, the model’s accuracy in the eating and lying categories dropped to 91.4% and 96.1%, respectively. Although the accuracy for the activity category remained stable, the mAP@0.5 decreased to 90.9%, which was inferior to the detection accuracy observed with other learning rates.

### 3.7. Performance Comparison of Different Object-Detection Models

**Figure 15:**
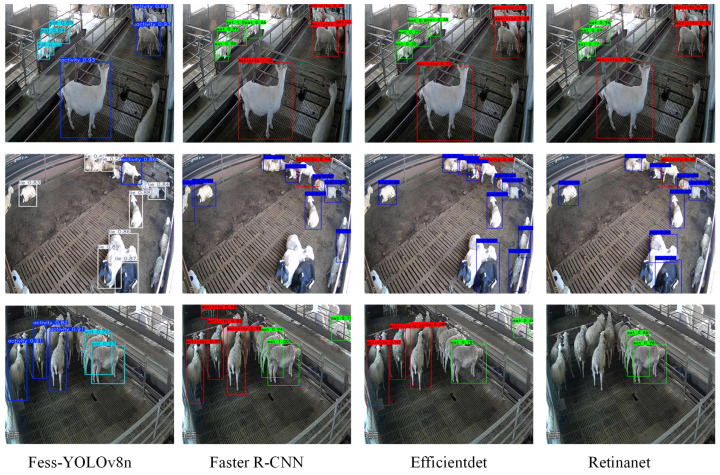
Recognition results for classical object detection models.

**Figure 16:**
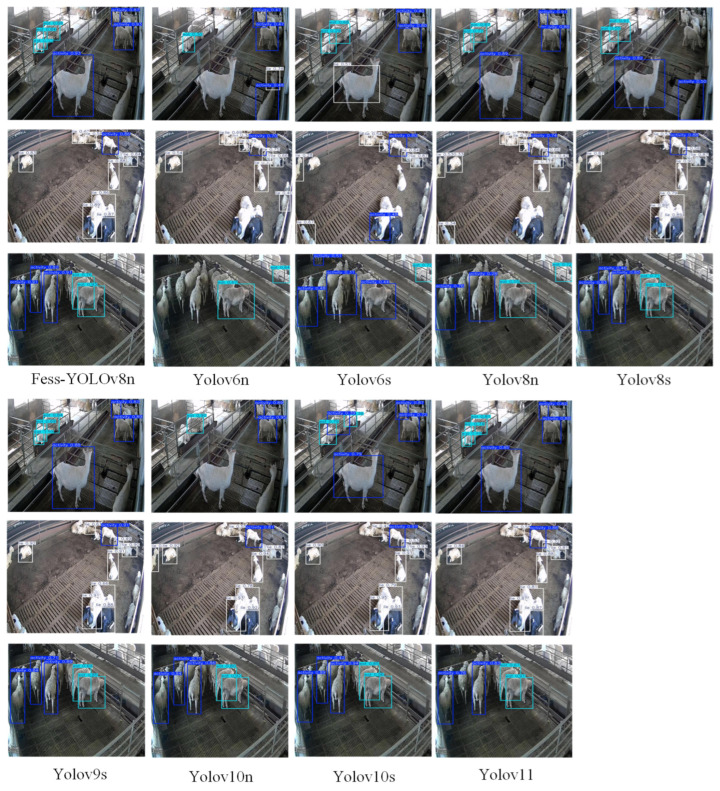
Recognition results for YOLO series object detection models.

To further validate the effectiveness of the Fess-YOLOv8n sheep behavior recognition model, comparative experiments were conducted with several traditional models, including Faster R-CNN, EfficientDet, RetinaNet, and versions of the YOLO series. The parameter settings during the training process were kept consistent. The comparison of the performances of the classical models in sheep behavior recognition is shown in Table 6 and Figure 15, while the comparison of the YOLO series is shown in Table 7 and Figure 16.

Based on the data presented in Table 6, it can be observed that the Fess-YOLOv8n model strikes a balance between high accuracy and lightweight design for sheep behavior recognition, achieving mAP@0.5 of 91.6%, a weight file size of 5.13 MB, and parameter size 3.69 M. In contrast, traditional object detection models such as Faster R-CNN, EfficientDet, and RetinaNet perform relatively poorly on this task. Specifically, the mAP@0.5 of Faster R-CNN was 82.63%, while both EfficientDet and RetinaNet failed to surpass 80%. Furthermore, these conventional models require substantial computational resources and storage space, with Faster R-CNN’s weight file and parameter size reaching 108 MB and 28.3 M, respectively. Moreover, Fess-YOLOv8n demonstrates significant advantages in both P and R, achieving values of 93% and 89.01%, respectively. While Faster R-CNN achieves a similar Precision of 90.63%, its Recall of 58.26% is considerably lower. From the comparison shown in Table 7, it was apparent that Fess-YOLOv8n demonstrated superior performance over the YOLO series models (YOLOv6n, YOLOv6s, YOLOv8n, YOLOv8s, YOLOv9s, YOLOv10n, YOLOv10s, and YOLOv11) by an improvement of 3% to 5% in mAP@0.5. Additionally, Fess-YOLOv8n demonstrated significantly lower parameter size compared to other YOLO versions, with reductions of 3.15 MB, 26.17 MB, 2.56 MB, 16.33 MB, 9.4 MB, 0.35 MB, 10.63 MB, and 0.08 MB, respectively. As a further point, Fess-YOLOv8n demonstrated significant advantages in both P and R. Although Fess-YOLOv8n did not show a markedly higher P compared to other models in the YOLO series, it achieved a notably higher R of 89.01%. To be specific, while models such as YOLOv8s and YOLOv9s achieved slightly higher *p*-values of 95.8% and 94.2%, respectively, they fell short in R, with values of 83.2% and 81.53%.

As shown in Figure 15, Faster R-CNN and RetinaNet perform well in non-dense environments but still experience missed detections in dense settings. The EfficientDet model suffers from low confidence scores, which affects its detection accuracy and reliability. In contrast, FESS-YOLOv8 demonstrates strong detection capabilities across various environmental conditions, both misdetections and missed detections. From the results in Figure 16, it is evident that the YOLOv6n and YOLOv6s models suffer from significant false detections and missed detections, resulting in lower detection accuracy. In comparison, the YOLOv8n model performs better than the YOLOv6 series but still encounters missed detections in dense sheep environments, and its bounding boxes are somewhat loose and do not fully fit the sheep. Although YOLOv8s, YOLOv10n, and YOLOv10s show improved detection performance compared to YOLOv6n, YOLOv6s, and YOLOv8n, they still exhibit missed and false detections when dealing with occluded sheep. YOLOv9s and YOLOv11 do not exhibit misdetections or missed detections, but their confidence scores for detecting sheep are relatively lower compared to FESS-YOLOv8.

## 4. Discussion

In this study, the Fess-YOLOv8n model significantly improved the accuracy of sheep behavior recognition, particularly in detecting dynamic activity behaviors. By modifying the YOLOv8n network, Fess-YOLOv8n demonstrated strong adaptability and accuracy in complex environments. This model provides livestock producers with an efficient and reliable tool to monitor sheep behavior in real time and assess their health status.

The design concept of the Fess-YOLOv8n model is mainly reflected in the following aspects. First, the C2f-Faster network structure is adopted, utilizing the FasterNet Block to optimize computational efficiency and achieve a lightweight design, which meets the real-time requirements of sheep behavior recognition. Second, to further enhance the model’s feature extraction capability, the EMA attention mechanism is integrated into the model. This design effectively addresses the limitations of traditional object detection methods in handling environmental interference and the randomness of sheep behavior in large-scale farming environments. Next, the SCSA module is introduced. This module combines channel and spatial dual attention mechanisms, allowing the model to more effectively extract key information from multiple dimensions, thereby improving the accuracy of behavior detection. Finally, to further optimize model performance, SCDown is introduced. This method reduces redundant calculations and parameters, lowering the model’s computational load while ensuring detection accuracy.

In addition, Fess-YOLOv8n has undergone detailed adjustments in model performance optimization. By adjusting the IoU threshold, the model can reduce false positive and false negative rates, further enhancing recognition accuracy. Meanwhile, the adjustment of the learning rate allows the model to converge more quickly during training while maintaining high accuracy.

While we have successfully developed the Fess-YOLOv8n model for sheep behavior detection, it still has some limitations. First, the dataset used in the current study is primarily sourced from artificial farming environments, and the sample size is relatively limited. Its generalizability and robustness in natural environments still need further validation. To enhance the model’s adaptability and generalization, future research could focus on expanding the dataset and including samples from various environmental conditions, such as different lighting conditions throughout the day and night. This would improve the model’s performance in a wider range of environments and further enhance its stability and accuracy in real-world applications. In addition, single-modal visual data may not fully capture the behavior characteristics of sheep. Future work could explore integrating multimodal data (such as sound and environmental monitoring data) for more precise behavior recognition.

## 5. Conclusions

With the rapid development of precision livestock farming, artificial intelligence, and deep learning technologies, livestock behavior monitoring has become increasingly important in animal husbandry. Efficient and accurate behavior recognition plays a crucial role in assessing the physiological health of livestock, while also offering a solid foundation for the scientific management of large-scale, automated farming systems. In sheep behavior recognition, the proposed Fess-YOLOv8n unsupervised detection model achieves an effective balance between lightweight design and high accuracy. Through improvements and comparative analysis, the following conclusions are drawn:

1. The Fess-YOLOv8n model utilizes the EMA structure, which significantly enhances the model’s ability to extract key information. The SCSA module improves the model’s feature extraction capabilities for sheep behavior, further enhancing its recognition accuracy. The C2f-Faster and SCDown modules notably reduce the model’s computational complexity and parameter count, achieving a lightweight design and improving detection speed. Experimental results show that the Fess-YOLOv8n model effectively recognizes sheep behavior, achieving mAP@0.5 of 91.4%, with a minimal weight file size of 5.13 MB.

2. Experimental results indicate that when the IoU threshold range is between 0.45 and 0.7, and the learning rate is 0.1, the mAP@0.5 of Fess-YOLOv8n reaches a peak of 91.6%, with the lowest false negative and false positive rates.

In summary, the Fess-YOLOv8n model is capable of quickly and accurately recognizing three distinct behaviors of sheep while maintaining a low false negative rate and false positive rate. Its efficient and precise characteristics not only provide crucial technical support for sheep behavior analysis and health management but also offer a solid foundation for the scientific management of sheep farming. In future work, we will expand the dataset by increasing samples from different environmental conditions, including those recorded in low-light or night-time settings, to better reflect real-world scenarios. Additionally, we will explore the integration of multimodal data to further enrich the dataset, improve model performance, and enhance its stability and effectiveness in real-world applications. This will enable the model to be applied to continuous, round-the-clock monitoring, ensuring its adaptability and robustness in varied environmental conditions.

## Figures and Tables

**Table 1:** Categories and labels of sheep behavior.

ID	Behavior Category	Behavior Description	Label	Label Count
1	activity	The sheep moves its body and limbs, with its head not touching the ground	activity	3575
2	eating	The sheep stands next to the feeding trough, with its head lowered into the trough	eat	5721
3	lying	The sheep sits on its hind legs, with its abdomen resting on the ground	lie	2441

**Table 2:** Results of ablation experiments for different improved models.

Experiment Name	Modified Model Name				GFLOPs	Weight	Parameters	mAP@0.5	AP		
	C2f-Faster	EMA	SCSA	SCDown					Activity	Eating	Lying
A1					8.20	7.69	2.56	86.90	76.70	91.00	92.90
A2	√				6.40	4.60	2.31	86.80	77.60	91.30	91.60
A3	√	√			6.50	5.95	2.31	88.30	77.90	92.70	94.30
A4	√	√	√		16.90	5.97	4.01	90.80	85.20	91.10	96.10
A5	√	√	√	√	16.60	5.13	3.69	91.40	85.40	92.20	96.50

Note: √ represents the parts of the model that have been added or modified. A1 represents the base YOLOv8n model; A2 represents A1 + C2f-Faster structure; A3 represents A2 + EMA structure; A4 represents A3 + SCSA structure; A5 represents A4 + SCDown, which is the final Fess-YOLOv8n model. The unit for weight file size is MB, the unit for parameter count is M, the unit for GFLOPs is operations, and the units for mAP@0.5 and AP are percentage (%).

**Table 3:** Comparison of Fess-YOLOv8n Performance at different IoU thresholds.

IoU Threshold	mAP@0.5	Activity		Eating		Lying	
		False Positive Rate	False Negative Rate	False Positive Rate	False Negative Rate	False Positive Rate	False Negative Rate
0.2	87.6	2	10	1	21	0	3
0.25	88.8	2	9	1	17	0	3
0.3	89.8	2	9	1	13	0	3
0.35	90.7	2	8	1	10	0	3
0.4	91.3	2	8	1	8	0	3
0.45	91.4	2	8	1	7	0	3
0.5	91.4	2	8	1	7	0	3
0.55	91.4	2	8	1	7	0	3
0.6	91.4	2	8	1	7	0	3
0.65	91.4	2	8	1	7	0	3
0.7	91.4	2	8	1	7	0	3
0.75	91.3	2	8	1	7	0	3

Note: The units for mAP@0.5, false positive rate, and false negative rate are all in percentage (%).

**Table 4:** Comparison of Fess-YOLOv8n under different optimizers.

Optimizer	mAP@0.5	AP		
		Activity	Eating	Lying
Adam	91.4	85.4	92.2	96.5
Nadam	80.4	70.9	81.2	89.1
Radam	84.6	75.4	89.0	89.4
Adamax	90.1	83.4	91.7	95.2
SGD	92.0	87.4	92.3	96.3

Note: The units of mAP@0.5, AP are all in percentage (%).

**Table 5:** Comparison of Fess-YOLOv8n under different learning rates.

Learning Rate	mAP@0.5	AP		
		Activity	Eating	Lying
0.1	91.6	85.7	92.2	96.7
0.01	91.4	85.4	92.2	96.5
0.001	90.9	85.3	91.4	96.1

Note: The units of mAP@0.5, AP are all in percentage (%).

**Table 6:** Performance comparison of classical object detection models.

Model	Weight	Parameters	P	R	mAP@0.5	AP		
						Activity	Eating	Lying
Fess-YOLOv8n	5.13	3.69	93.00	89.01	91.60	85.70	92.20	96.70
Faster R-CNN	108.00	28.30	90.63	58.26	82.63	75.00	90.00	84.00
EfficientDet	15.00	3.92	79.57	56.93	72.67	65.00	81.00	72.00
RetinaNet	139.00	36.20	78.99	69.60	78.72	72.00	86.00	78.00

**Table 7:** Performance comparison of YOLO series object detection models.

Model	Weight	Parameters	P	R	mAP@0.5	AP		
						Activity	Eating	Lying
Fess-YOLOv8n	5.13	3.69	93.00	89.01	91.60	85.70	92.20	96.70
YOLOv6n	8.28	4.23	91.60	83.33	86.50	76.10	90.60	92.90
YOLOv6s	31.30	16.30	94.80	83.21	87.50	76.70	91.90	94.00
YOLOv8n	7.69	2.56	93.70	85.41	86.90	76.70	91.00	92.90
YOLOv8s	21.46	9.31	95.80	83.20	87.60	75.60	92.50	94.70
YOLOv9s	14.53	7.28	94.20	81.53	88.40	77.80	92.10	95.20
YOLOv10n	5.48	2.71	93.90	85.19	87.50	77.80	91.80	92.80
YOLOv10s	15.76	8.06	96.80	84.02	88.30	78.90	91.80	94.20
YOLOv11	5.21	2.59	90.00	83.73	87.30	78.70	91.00	92.20

Note: The unit of the weight file size is MB, the unit for parameter count is M, and the units for P, R, mAP @0.5 and AP are all in percentage (%).

## Data Availability

The data used in this study were sourced from a private farm and are non-public. Due to privacy protection and commercial confidentiality, the data are not made available to the public.
